# The antibacterial activity of berberine against *Cutibacterium acnes*: its therapeutic potential in inflammatory acne

**DOI:** 10.3389/fmicb.2023.1276383

**Published:** 2024-01-05

**Authors:** Luyao Sun, Qian Yu, Fu Peng, Chen Sun, Daibo Wang, Lin Pu, Fang Xiong, Yuncai Tian, Cheng Peng, Qinmei Zhou

**Affiliations:** ^1^State Key Laboratory of Characteristic Chinese Medicine Resources in Southwest China, Chengdu University of Traditional Chinese Medicine, Chengdu, China; ^2^School of Basic Medicine, Chengdu University of Traditional Chinese Medicine, Chengdu, China; ^3^Key Laboratory of Drug-Targeting and Drug Delivery System of the Education Ministry and Sichuan Province, Sichuan Engineering Laboratory for Plant-Sourced Drug and Sichuan Research Center for Drug Precision Industrial Technology, West China School of Pharmacy, Sichuan University, Chengdu, China; ^4^Shanghai Zhizhenzhichen Technologies Co., Ltd., Shanghai, China; ^5^Innovative Institute of Chinese Medicine and Pharmacy, Chengdu University of Traditional Chinese Medicine, Chengdu, China

**Keywords:** berberine, *Cutibacterium acnes*, antibacterial activity, peptidoglycan synthesis, anti-inflammatory, inflammatory cytokines

## Abstract

*Cutibacterium acnes* (*C. acnes*) is a major pathogen implicated in the evolution of acne inflammation. Inhibition of *C. acnes*-induced inflammation is a prospective acne therapy strategy. Berberine (BBR), a safe and effective natural ingredient, has been proven to exhibit powerful antimicrobial and anti-inflammatory properties. However, the antimicrobial effect of BBR against *C. acnes* and its role in *C. acnes*-mediated inflammatory acne have not been explored. The objective of this investigation was to assess the antibacterial activity of BBR against *C. acne*s and its inhibitory effect on the inflammatory response. The results of *in vitro* experiments showed that BBR exhibited significant inhibition zones against four *C. acnes* strains, with the minimum inhibitory concentration (MIC) and minimum bactericidal concentration (MBC) in the range of 6.25–12.5 μg/mL and 12.5–25 μg/mL, respectively. On the bacterial growth curve, the BBR-treated *C. acnes* exhibited obvious growth inhibition. Transmission electron microscopy (TEM) images indicated that BBR treatment resulted in significant morphological changes in *C. acnes*. High-content imaging analysis further confirmed that BBR could effectively inhibit the proliferation of *C. acnes*. The disruption of cell wall and cell membrane structure by BBR treatment was preliminary confirmed according to the leakage of cellular contents such as potassium (K^+^), magnesium (Mg^2+^), and alkaline phosphatase (AKP). Furthermore, we found that BBR could reduce the transcript levels of genes associated with peptidoglycan synthesis (*murC*, *murD*, *mraY*, and *murG*). Meanwhile, we investigated the modulatory ability of BBR on *C. acnes*-induced skin inflammation in mice. The results showed that BBR effectively reduced the number of *C. acnes* colonized in mice’s ears, thereby alleviating ear swelling and erythema and significantly decreasing ear thickness and weight. In addition, BBR significantly decreased the levels of pro-inflammatory cytokines IL-6, IL-1β, and TNF-α in auricular tissues. These results suggest that BBR has the potential to treat inflammatory acne induced by *C. acnes*.

## 1 Introduction

Acne is a prevalent skin condition, affecting about 85% of adolescents globally (Lynn et al., 2016; Picardo et al., 2022). The mechanisms of this disease are quite sophisticated and are not yet completely explained (Knutsen-Larson et al., 2012). Generally, it’s recognized that abnormal proliferation of *Cutibacterium acnes* (*C. acnes*) is an important cause of the inflammatory reaction, leading to the formation of acne (Beylot et al., 2014; Dréno et al., 2018). Over the years, a variety of antibiotics have been extensively used in the therapy of acne, including clindamycin, macrolides, and tetracyclines (Adler et al., 2017). The emergence and spread of antimicrobial strains of *C. acnes* has become a major problem in contemporary dermatology due to the repeated use of antimicrobials as monotherapy over a long period of time (Aslan Kayiran et al., 2020). Currently, the main treatments available for acne are isotretinoin, azelaic acid, and retinoids. Despite their effectiveness on acne, prolonged use could possibly induce adverse reactions such as local dryness, redness, irritation, and peeling (Habeshian and Cohen, 2020). Consequently, natural pharmaceutical active ingredients are an important source of avenues for new drug discovery that play an important role in the development of innovative drugs for acne.

Berberine (BBR), an isoquinoline alkaloid (5,6-dihydroxybenzoquinone derivative), is a secure and effective natural ingredient that can be extracted from a wide range of medicinal plants, such as *Coptis chinensis*, *Berberis vulgaris*, and *Hydrastis canadensis* (Warowicka et al., 2020). It has been revealed that BBR has multiple pharmacological characteristics, including anti-cancer, anti-inflammatory, antimicrobial, cardioprotective, and neuroprotective activities (Feng et al., 2019; Song et al., 2020; Shou and Shaw, 2022; Xiong et al., 2022). Notably, BBR has also received great attention for its high ability against various pathogenic microorganisms (Li et al., 2018; Jamshaid et al., 2020; Warowicka et al., 2020), but its suppressive effect on *C. acnes* has not been reported. With the aim of discovering effective agents for acne, we investigated the antimicrobial effect of BBR against *C. acnes* and its role in *C. acnes*-mediated inflammatory acne for the first time. According to the findings of this study, there may be a new therapeutic option for acne.

## 2 Materials and methods

### 2.1 Medicine

**FIGURE 1:**
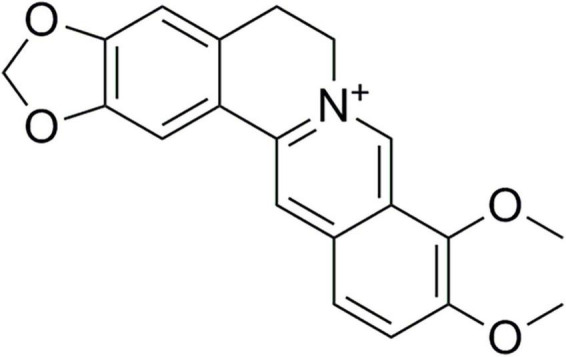
The chemical structure of berberine.

Berberine (C_20_H_18_NO_4_, purity ≥ 98.0%, Lot No. AZ21090715) was obtained from Chengdu Alfa Biotechnology Co., Ltd. (Chengdu, China). The chemical structure of BBR was shown as Figure 1. Doxycycline (purity ≥ 98.0%, Lot No. D14332) was obtained from Frontier Scientific, Inc. (USA).

### 2.2 Microbial strain and growth condition

*Cutibacterium acnes* (ATCC 11827) was acquired from the Guangdong Microbial Culture Collection Center (Guangzhou, China). *C. acnes* (BNCC 330605, BNCC 252396, and BNCC 192214) was acquired from the Beijing BeNa Culture Collection (Beijing, China). The strain was incubated in Brain-Heart Infusion (BHI) broth (Beijing Solarbio Science and Technology Co., Ltd., Beijing, China) medium under anaerobic conditions at 37°C. When the bacteria had grown to the logarithmic growth stage, the inoculum concentration was adjusted with a turbidity of 0.5 McFarland’s standard (1.5 × 10^8^ CFU/mL). The bacterial cultures were diluted 1:100 for a final inoculum of 1.5 × 10^6^ CFU/mL.

### 2.3 Determination of the inhibition zones

The procedure for exploring the antibacterial activity of BBR was performed by the previously reported agar diffusion method with a few modifications (Wang et al., 2020). A total of 100 μL of the bacterial suspension (1.5 × 10^6^ CFU/mL) was evenly spread on BHI agar plates and dried naturally. Subsequently, the blank discs (6 mm in diameter) covered with 10 μL BBR (10 mg/mL) were laid flat on the surface of the BHI agar plates, with 0.1% DMSO as the blank control and doxycycline (10 μg/disc) as the positive control. The inhibition zones were measured after 72 h of anaerobic incubation at 37°C in a thermostatic incubator.

### 2.4 Determination of MIC and MBC

The two-fold dilution method was employed to determine the MIC and MBC of BBR against the four *C. acnes* strains (Poomanee et al., 2018). Briefly, the BBR was serially diluted in 96-well plates, and then the bacterial suspension was added to ensure that the final concentrations of the BBR were 50, 25, 12.5, 6.25, 3.13, 1.56, 0.78, and 0.39 μg/mL, respectively. The final inoculum concentration of the bacterial suspension was 1.5 × 10^6^ CFU/mL. A total of 0.1% DMSO was used as a control, and doxycycline was used as a positive control. The plates were incubated anaerobically for 72 h at 37°C in a permanent incubator. The lowest dilution concentration of clarified sterile growth was used as the MIC to observe and record the results. To determine the MBC of BBR, 5 μL of supernatant was removed from the clarified wells, inoculated onto BHI agar plates, and then continued to be anaerobically cultured for 72 h at 37°C in a thermostatic incubator. The number of colonies was observed, and the concentration of BBR in the corresponding well with no colony growth was recognized as MBC.

### 2.5 Bacterial growth curve determination

One bacteria strain, *C. acnes* (ATCC 11827), was selected for further study of the antimicrobial effect of BBR on *C. acnes* in depth. The growth curves were detected with previous experimental methods to verify the antimicrobial activity of BBR (Cao et al., 2019). In a 96-well plate, different concentrations of BBR were added to each well, followed by the bacterial suspension (with a final inoculum concentration of 1.5 × 10^6^ CFU/mL) to give a terminal concentration of BBR at 0.25 MIC, 0.5 MIC, MIC, and 2 MIC. A total of 0.1% DMSO was set as the control. The cultures were cultivated anaerobically in a 37°C incubator for 72 h, and the absorbance values at 600 nm were recorded every 4 h with a FlexStation 3 multi-mode microplate reader (Molecular Devices, USA). Eventually, the bacterial growth curves were plotted.

### 2.6 Morphological observation by TEM

The experimental method for observing changes in the morphology and structure of *C. acnes* (ATCC 11827) using transmission electron microscopy (TEM) was the same as described in the previous study, with several improvements (Li et al., 2014). *C. acnes* bacterial suspension (1.5 × 10^6^ CFU/mL) was treated with MIC concentrations of BBR. The cultures were incubated in a thermostatic shaker at 37°C with shaking at 150 rpm under anaerobic conditions for 72 h. A total of 0.1% DMSO served as a control. The bacterial precipitate was obtained by centrifugation at 6,000 *g* for 10 min at 4°C and then washed three times with PBS (0.1 M, pH 7.4). The bacteria were pre-fixed with 3% glutaraldehyde and then re-fixed with 1% osmium tetroxide. This was followed by three rounds of dehydration with a gradient of graded acetone solutions (30, 50, 70, 80, 90, and 100%), then permeabilization, embedding, sectioning, and staining. Finally, the samples were examined with a TEM (JEM-1400FLASH, JEOL, Japan).

### 2.7 Detection of bacterial proliferation by a high-content imaging system

Proliferation of *C. acnes* (ATCC 11827) was detected using high-content imaging as described in previous studies, with some modifications (Portella et al., 2021). The bacterial suspension (1.5 × 10^6^ CFU/mL) was inoculated into 96 wells pre-coated with 0.1% poly-L-lysine (PLL). And the BBR solution was added and diluted in a gradient of BHI medium to give a final concentration of 0.5 MIC, MIC, and 2 MIC, and 0.1% DMSO was utilized as a control. Three replicated wells were set up for each group. Subsequently, the plates were anaerobically cultivated for 24 h in an incubator at 37°C. The bottom of the well plate was rinsed three times with PBS after gently aspirating the culture solution and fixed by adding 30 μL of 4% paraformaldehyde for 30 min. The wells were gently aspirated again and incubated for 30 min with 30 μL of Hoechst 33342 staining solution (Shanghai Yuanye Bio-Technology Co., Ltd., Shanghai, China). The plate was then washed three times with PBS, and finally the fluorescence intensity was observed and analyzed with the Image Xpress Micro Confocal High-Content Imaging System (Molecular Devices, US).

### 2.8 Measurement of the leakage of K^+^, Mg^2+^, and AKP

A further study of the integrity of the bacterial cell wall was conducted by examining the leakage of K^+^, Mg^2+^, and AKP in BBR-treated *C. acnes* (ATCC 11827) (Guo et al., 2021). *C. acnes* was cultured to logarithmic phase and then treated with BBR so that the final concentration of BBR was 0.5 MIC, MIC, and 2 MIC. All cultures were incubated anaerobically in a shaker for 12 h (150 rpm at 37°C). A total of 0.1% DMSO was taken as a control. The leakage of K^+^, Mg^2+^, and AKP from the cultures was measured by the K^+^, Mg^2+^, and AKP assay kits (Nanjing Jiancheng Bioengineering Institute, Jiangsu, China) as directed by the manufacturer.

### 2.9 qRT-PCR analysis

To analyze the transcription level of mRNA, quantitative real-time PCR (qRT-PCR) analysis was performed in line with previous studies (Qin et al., 2023). *C. acnes* (ATCC 11827) was incubated to the logarithmic stage before being exposed to BBR at a concentration of MIC. A total of 0.1% DMSO was set as the control group. Total RNA was obtained from *C. acnes* with Trizol reagent (Beyotime Biotechnology, Shanghai, China), and the concentration was ascertained through photometric measurement. To synthesize cDNA from RNA, a High-Capacity cDNA Reverse Transcription Kit (Thermo Fisher Scientific, USA) was utilized as directed by the manufacturer. The expressions of peptidoglycan synthesis-related genes (*murC*, *murD*, *mraY*, and *murG*) were tested by qRT-PCR using PowerUp™ SYBR™ Green Master Mix (Thermo Fisher Scientific, USA). The samples were normalized with *gyrB* (endogenous control). The results were analyzed by the 2^–ΔΔCt^ method. Table 1 lists the primers that were utilized.

**TABLE 1:** Primers used for the study.

Genes	Primer direction	Sequence (5′-3′)
*gyrB*	Forward	CTGCCCCAAGTTAACCCGAT
	Reverse	TGGCATTGCGCTGAATGAAC
*murC*	Forward	CTTGGTGGGGGAGAAGCATT
	Reverse	GACACGAAGCCGTCGAAGTA
*murD*	Forward	ACGACTACCCAGATGACCGA
	Reverse	TGGACAGCTCCACTGCAAAA
*mraY*	Forward	ATTGTCTCCGTACTGCTGGC
	Reverse	CCGATGAAACCCAATCCCGA
*murG*	Forward	TTGCGTTCACAGATCACCGA
	Reverse	TCTGCAAGTAGACGTGTCCG

### 2.10 Effect of BBR on *C. acnes*-induced inflammation *in vivo*

Eight-week-old male ICR mice were procured from Chengdu Dossy Experimental Animals Co., Ltd. All animal experimental procedures followed animal ethical rules and were authorized by the Chengdu University of Traditional Chinese Medicine committee (No. 2022-67). The anti-inflammatory impact of BBR *in vivo* was assessed using an intradermal injection method (Huang et al., 2015). In the initial experiment, 20 μL of BBR (up to a maximum dose of 0.5 mg/mL) was injected intradermally into the ears of mice, and no significant skin irritation occurred (data not shown). As a result, doses of 0.5, 0.25, and 0.125 mg/mL were used for the following trials. ICR mice were randomly divided into 6 groups (*n* = 6), including control, model, doxycycline (0.05 mg/mL), and BBR intervention groups (0.5, 0.25, and 0.125 mg/mL). Acne infection and inflammation were induced by the intradermal injection of 20 μL of *C. acnes* (ATCC 11827, 1.5 × 10^6^ CFU/mL) into the mouse pinna. The control group was given the same quantity of PBS (20 μL). Following the injection of *C. acnes*, 20 μL of BBR was delivered into the BBR intervention group at the same location, while 20 μL of PBS was administered into the control and model groups.

After 24 h of bacterial injection, the thickness of the auricle was measured with a micro-vernier caliper. Mice were executed with an overdose of pentobarbital, and ear discs 8.0 mm in diameter were perforated and weighed. For histological observation, the ears were stained with hematoxylin and eosin before being observed under the microscope for inflammatory cell infiltration. The auricular tissues were homogenized with sterile PBS and serially diluted on BHI agar plates. The colonies of bacteria were analyzed after anaerobic incubation at 37°C for 72 h. ELISA kits (Elabscience Biotechnology Co., Ltd., Wuhan, China) were employed to measure IL-6, IL-1β, and TNF-α levels in ear tissues.

### 2.11 Statistical analysis

The data were represented as the mean ± SD, and differences among multiple groups were analyzed through a one-way ANOVA using SPSS 22.0. It was considered statistically significant if there was a difference between groups at *p* < 0.05 or 0.01.

## 3 Results

### 3.1 Determination of the inhibition zones

In order to explore the inhibitory effect of BBR on *C. acnes*, we performed an agar diffusion assay. As shown in Table 2, the inhibition zones of BBR against ATCC 11827, BNCC 330605, BNCC 252396, and BNCC 192214 were 35.27 mm, 35.10 mm, 34.77 mm, and 32.07 mm, respectively, which indicated that BBR can inhibit the growth of *C. acnes*. Doxycycline, a standard antibiotic, showed higher bactericidal activity against the four strains, whereas the control group (0.1% DMSO) did not exhibit any noteworthy zones of inhibition.

**TABLE 2:** The inhibition zones of BBR and doxycycline against four *C. acnes* strains.

*C. acnes*	The inhibition zones (mm)	
	BBR (100 μ g/disc)	Doxycycline (10 μ g/disc)
ATCC 11827	35.27 ± 0.35	40.80 ± 0.35
BNCC 330605	35.10 ± 0.26	40.67 ± 0.40
BNCC 252396	34.77 ± 0.21	40.57 ± 0.31
BNCC 192214	32.07 ± 0.35	37.60 ± 0.53

### 3.2 Determination of the MIC and MBC of BBR

MIC and MBC are important indicators of antibacterial activity for the drug. As shown in Table 3, it was observed that BBR exhibited bacteriostatic and bactericidal activity against the four *C. acnes* strains. The MIC values of BBR against *C. acnes* ranged from 6.25 to 12.5 μg/mL, and the MBC was in the range of 12.5–25 μg/mL. Meanwhile, the MIC and MBC of *C. acnes* to doxycycline were 0.31 μg/mL and 0.63 μg/mL, indicating that the results of this experiment were reliable. Hence, it is necessary to further explore the mechanism of BBR toward *C. acnes*.

**TABLE 3:** The MIC and MBC of BBR and doxycycline against four *C. acnes* strains.

*C. acnes*	BBR		Doxycycline	
	MIC (μ g/mL)	MBC (μ g/mL)	MIC (μ g/mL)	MBC (μ g/mL)
ATCC 11827	6.25	12.5	0.31	0.63
BNCC 330605	6.25	12.5	0.31	0.63
BNCC 252396	6.25	12.5	0.31	0.63
BNCC 192214	12.5	25	0.31	0.63

### 3.3 Effects of BBR on the growth curve

**FIGURE 2:**
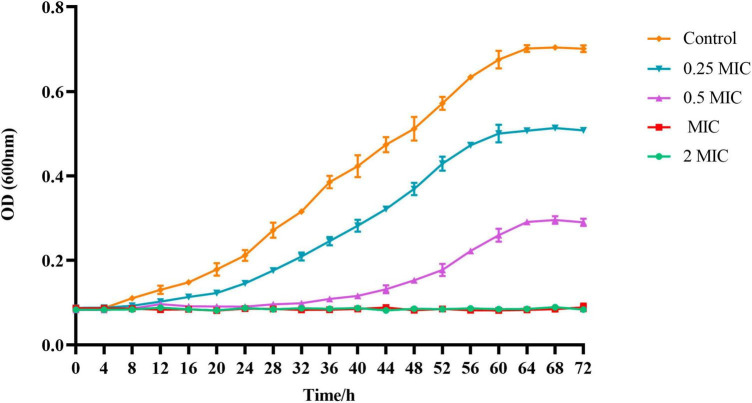
The absorbance values at 600 nm were recorded every 4 h up to 72 h using a microplate reader to obtain the growth curve of *C. acnes* (ATCC 11827) treated with BBR. For *C. acnes*, 2 MIC: 12.5 μg/mL, MIC: 6.25 μg/mL, 0.5 MIC: 3.125 μg/mL, 0.25 MIC: 1.56 μg/mL. Control: 0.1% DMSO.

The growth curve of *C. acnes* (ATCC 11827) was developed in order to further validate the antibacterial activity of BBR. In accordance with Figure 2, the OD_600_ of the control group of *C. acnes* started at 8 h and then increased rapidly from 12 h to 56 h. Then, the optical density increased gradually and remained stable. The highest densities were observed when the time reached 64 h, which is primarily due to the life cycle of the bacteria. The growth curve of *C. acnes* treated with BBR was significantly inhibited compared to the control group. The higher the concentration of BBR exposed, the stronger the inhibitory effect on *C. acnes*. The results of this experiment indicated that the growth of *C. acnes* could be completely inhibited when the concentration of BBR was over MIC, which reflected the high antimicrobial activity of BBR on *C. acnes*.

### 3.4 BBR alters the membrane and structure of *C. acnes*

**FIGURE 3:**
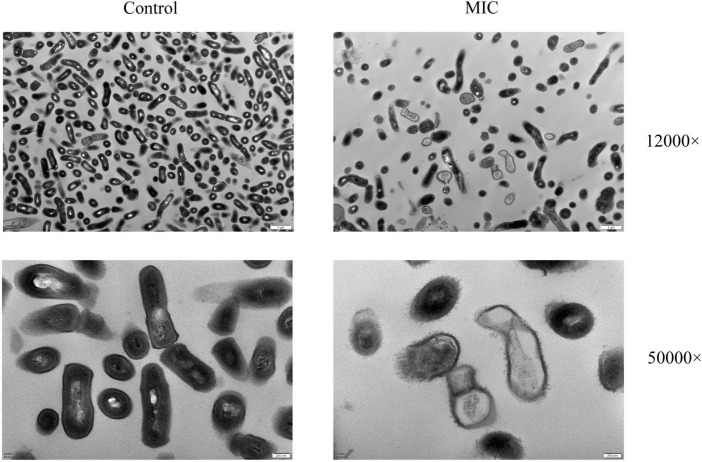
Images of the morphological influences of BBR on *C. acnes* (ATCC 11827) were obtained using TEM at 12,000 and 50,000 × magnifications, respectively. For *C. acnes*, MIC: 6.25 μg/mL. Control: 0.1% DMSO.

The changes in the morphology and ultrastructure of the bacteria can be directly reflected by TEM micrographs. To further confirm the antibacterial effect of BBR against *C. acnes* (ATCC 11827), TEM analysis was performed. As shown in Figure 3, untreated *C. acnes* had a smooth, rod-shaped surface with intact structure and uniform size and distribution. Meanwhile, significant morphological damage was caused by BBR treatment of *C. acnes*. A large number of bacterial cells were bent, sunken, and deformed, as well as rough and uneven surfaces. A bacterial rupture was also observed. The TEM analysis confirmed that BBR altered normal cell morphology and disrupted cell walls and membranes, thus inhibiting the growth of *C. acnes* and even killing it.

### 3.5 High-content imaging analysis

**FIGURE 4:**
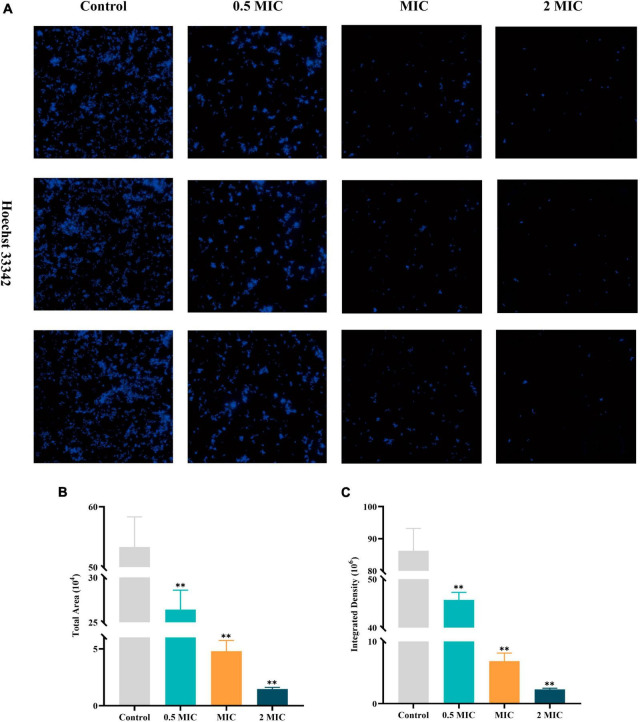
High-content imaging analysis of *C. acnes* (ATCC 11827) after BBR treatment. **(A)** Fluorescence image of *C. acnes* after Hoechst 33342 staining, obtained using a high-content imaging system. **(B,C)** Histogram of the total area and integrated density of *C. acnes*. The data are expressed as mean values ± SD. *n* = 3, **p < 0.01, versus the control. For *C. acnes*, 2 MIC: 12.5 μg/mL, MIC: 6.25 μg/mL, 0.5 MIC: 3.125 μg/mL. Control: 0.1% DMSO.

Hoechst 33342, a blue fluorescent dye that penetrates cell membranes, is commonly used for general nuclear staining. In this article, the impact of BBR on the proliferation of *C acnes* (ATCC 11827) was investigated by high-content imaging using fluorescence assays with Hoechst 33342 staining solution. *C. acnes* treated with BBR significantly reduced the total area and integrated density of fluorescence at dosages of 0.5 MIC, MIC, and 2 MIC (Figure 4). It was affirmed that BBR can effectively suppress the proliferation of *C. acnes*.

### 3.6 Effects of BBR on the leakage of cellular material

**FIGURE 5:**
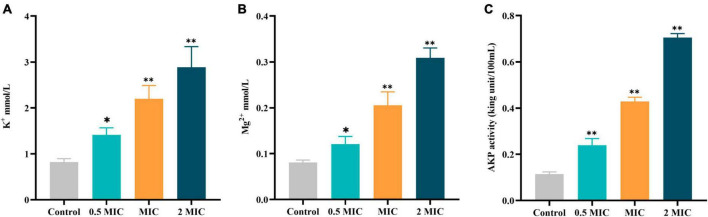
Effects of BBR on the leakage of cellular contents of *C acnes* (ATCC 11827). **(A)** K^+^ in the supernatant of *C. acnes*. **(B)** Mg^2+^ in the supernatant of *C. acnes*. **(C)** AKP in the supernatant of *C. acnes*. The data are expressed as mean values ± SD. *n* = 3, *p < 0.05, **p < 0.01, versus the control. For *C. acnes*, 2 MIC: 12.5 μg/mL, MIC: 6.25 μg/mL, 0.5 MIC: 3.125 μg/mL. Control: 0.1% DMSO.

Freeing up small molecules such as K^+^, Mg^2+^, and AKP also increases with the disruption of cellular structure. Therefore, any changes in the content of cellular contents could be regarded as an index of the structural integrity of the cell. As shown in Figure 5, the leakage of K^+^, Mg^2+^, and AKP was significantly increased in a dose-dependent pattern after exposure to BBR for 12 h as compared with the control group. The findings of this study suggest that BBR can destroy the integrity of cell walls and membranes, leading to leakage of cell contents.

### 3.7 Effect of BBR on the expression of genes related to peptidoglycan synthesis in *C. acnes*

**FIGURE 6:**
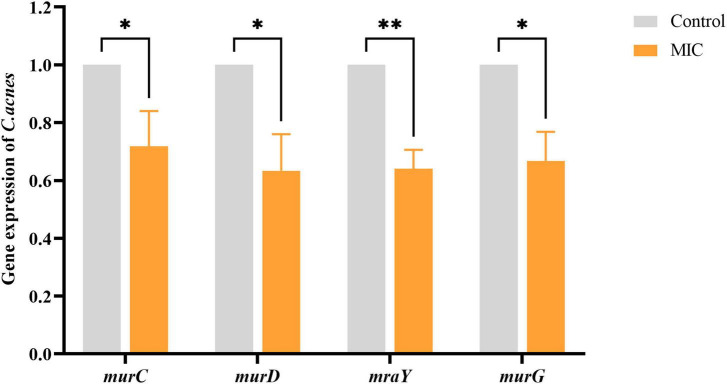
Effects of BBR on the expression of cell wall peptidoglycan synthesis-related genes. The data are expressed as mean values ± SD. *n* = 3, *p < 0.05, **p < 0.01, versus the control. For *C. acnes*, MIC: 6.25 μg/mL. Control: 0.1% DMSO.

To assess the effect of BBR on the cell wall synthesis-associated genes of *C. acnes* (ATCC 11827), qRT-PCR analysis was conducted. The results (Figure 6) showed that the expression of representative genes *murC* (coding UDP-N-acetylmuramate-L-alanine ligase), *murD* (coding UDP-N-acetylmuramoyl-L-alanine-D-glutamate ligase), *mraY* (coding phospho-N-acetylmuramoyl-pentapeptide-transferase), and *murG* (coding N-acetylglucosaminyl transferase) associated with cell wall PG synthesis were down-regulated to varying degrees when exposed to BBR at a concentration of MIC (*p* < 0.05 or 0.01).

### 3.8 Effect of BBR on *C. acnes*-induced inflammation *in vivo*

**FIGURE 7:**
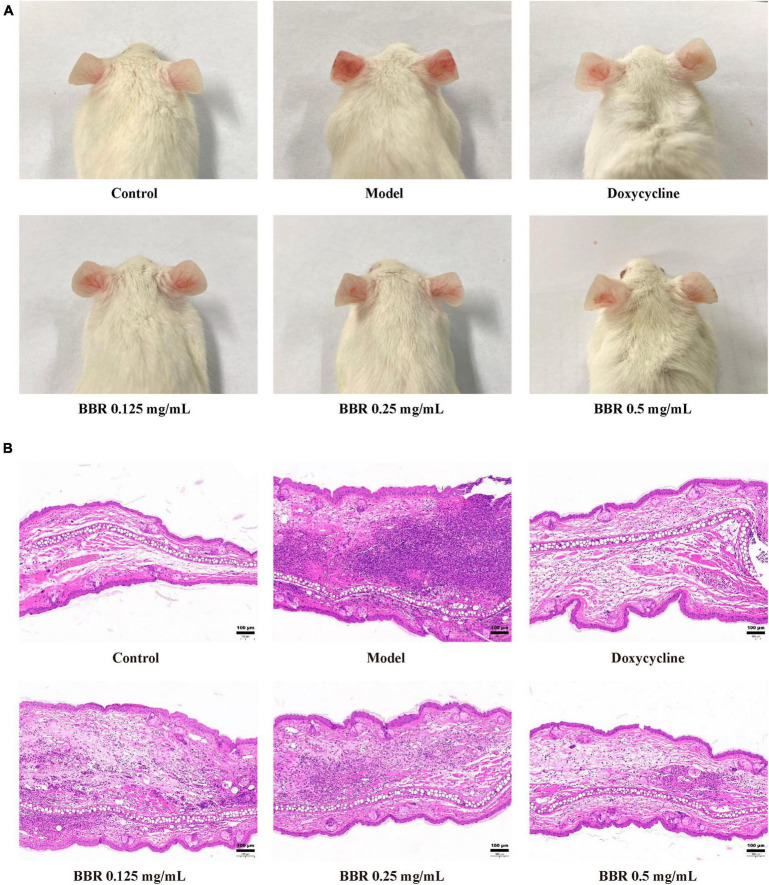
The inhibitory effect of BBR on the inflammatory response induced by *C. acnes* (ATCC 11827). **(A)** The representative picture of mice auricular inflammation. **(B)** The pathological changes in mice observed after hematoxylin and eosin staining. The scale bar represents 100 μm.

**FIGURE 8:**
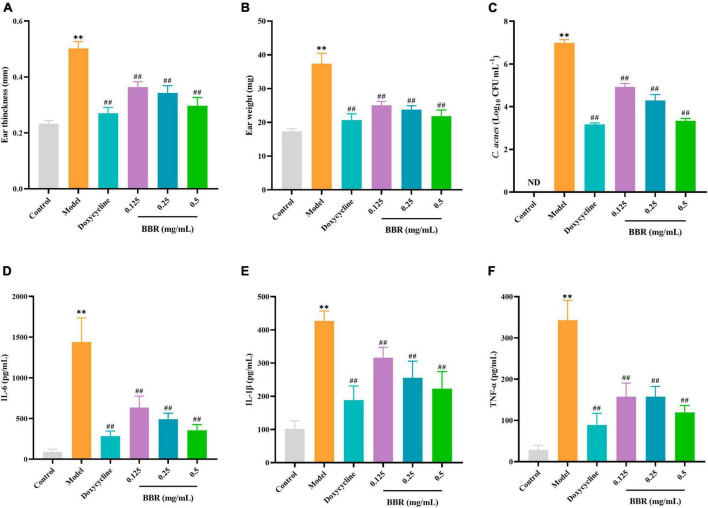
The evaluation of the inhibitory effect of BBR on *C. acnes*-induced inflammation in the mice ear. **(A,B)** Effect of BBR on ear thickness and ear weight in mice. **(C)** Effect of BBR on the number of *C. acnes* (ATCC 11827) colonizing the ear in mice ears. **(D–F)** Effects of BBR on the levels of IL-6, IL-1β, and TNF-α in auricular tissues, respectively. The data are expressed as mean values ± SD, *n* = 6, **p < 0.01, versus the control, ^##^*p* < 0.01, versus the model.

To investigate the antimicrobial and anti-inflammatory effects of BBR *in vivo*, an intradermal injection of *C. acnes* (ATCC 11827) was administered into the auricle of mice, which showed significant swelling and redness 24 h after the injection (Figure 7A). In the current study, the BBR intervention significantly ameliorated *C. acnes*-induced skin inflammation and reduced the increase in auricular thickness (Figure 8A), weight (Figure 8B), and the number of *C. acnes* colonizing the ear (Figure 8C) (*p* < 0.05 or 0.01). The histology results revealed that infusion of *C. acnes* resulted in a significant increase in the quantity of inflammatory cell infiltrates, which could be significantly reduced after BBR treatment (Figure 7B). The anti-inflammatory action of BBR *in vivo* was further assessed by measuring IL-6, IL-1β, and TNF-α levels in ear tissues. The expression levels of IL-6, IL-1β, and TNF-α were considerably increased in auricular tissues after injection, and these inflammatory factors were notably decreased after BBR treatment (Figures 8D–F) (*p* < 0.05 or 0.01). These data demonstrated that BBR effectively inhibited *C. acnes*-induced inflammation *in vivo*.

## 4 Discussion

Acne is a common chronic inflammatory skin disease with a high occurrence rate. If it is not treated properly, it is easy to leave scars, which will have a serious detrimental influence on the quality of life and psychosocial function of patients. Antibiotics are a successful option for treating acne in the case of microbial infections. However, the prolonged and widespread adoption of antibiotics has ultimately caused a high level of drug resistance globally (Abushaheen et al., 2020). And a rising number of studies have reported resistance to a variety of antibiotics in *C. acnes*, which limits its use as a therapy (Sardana et al., 2015; Adler et al., 2017). In order to avoid drug resistance, natural extracts, including essential oils and total flavonoids, have been widely used in many cosmetics or supplements to promote acne in recent years (Mohammad et al., 2018; Nurzyńska-Wierdak et al., 2022). As herbal skincare has a long history in skincare, it gains more and more attention and recognition.

Berberine is a well-known anti-infective agent that originated in traditional Chinese medicine. It has been demonstrated to exhibit remarkable antimicrobial potential on methicillin-resistant *Staphylococcus aureus* (Zhang et al., 2020), *Streptococcus pyogenes* (Du et al., 2020), and *Enterococcus faecalis* (Chen et al., 2016), *Escherichia coli* (Sun et al., 2019), *Salmonella typhimurium* (Xu et al., 2021), and *Pseudomonas aeruginosa* (Mangiaterra et al., 2021). Although several studies have investigated the possible antibacterial mechanism of BBR, no study has yet described its effects on *C. acnes*. In this article, the antibacterial activity and mechanism of BBR against *C. acnes* were researched. The results showed that BBR possessed markable inhibitory activity against four *C. acnes* strains (zone of inhibition ranges from 32.07 to 35.27 mm, MIC and MBC in the range of 6.25–12.5 μg/mL and 12.5–25 μg/mL, respectively). The growth process of *C. acnes* was tested using standard optical density measurements. It was found that all groups treated with BBR inhibited the growth of *C. acnes* in comparison to the control group. Then, we further investigated the antibacterial mechanism of BBR. Changes in TEM micrographs can directly reflect the morphology and ultrastructure of the bacterium. The results showed that *C. acnes* in the control group presented normal morphological features. They showed smooth surfaces, clear boundaries, and homogeneous cytoplasmic areas. As expected, significant microscopic morphological changes in *C. acnes* were visualized in BBR treatment. The shape of *C. acnes* became irregular, and the cell wall and cell membrane were damaged, leading to blurred boundaries, leakage of cytoplasmic contents, and disruption of cytoplasmic homogeneity. These data indicated that BBR has a strong damaging and inhibitory effect on *C. acnes*. High-content imaging analysis further confirmed that BBR could effectively inhibit the proliferation of *C. acnes*.

Studies have shown that BBR can interact with many targets in the bacterial cell wall and membrane, disrupting the structure of the cell wall and membrane and hindering the synthesis process (Zorić et al., 2017; Zhang et al., 2020). Therefore, we hypothesize that the anti-*C. acnes* effect of BBR may be related to the breakdown of the cell wall and membrane and the release of intracellular substances. It is well known that the cell wall of bacteria has the function of providing protection to the bacteria and transporting substances. In addition to affecting growth, it also affects the ability of the cells to resist exogenous factors (Dmitriev et al., 2005). The AKP enzyme is usually positioned between the cell wall and membrane and can only be detected in the extracellular environment when the bacterial cell wall is disrupted. Therefore, AKP is typically used as an index of cell wall integrity (Gu et al., 2023). In this study, the level of AKP in the extracellular environment of *C. acnes* was notably increased after BBR treatment, indicating that BBR disrupted the cell wall of *C. acnes*, which led to the increasing permeability of the cell wall and leakage of AKP outside the cell.

The cell membrane acts as a selective permeability barrier, separating cells from their external environment, which is essential for maintaining a stable internal environment and promoting metabolic activity (Strahl and Errington, 2017). There is growing evidence that the anti-bacterial mechanisms of natural compounds are involved in the breakage of the cell membrane and the spillage of intracellular components (Meng et al., 2016; Vartika et al., 2022). When the cell membrane of bacteria is disrupted, some molecules within the cell tend to escape, including some large molecules (nucleic acids, proteins, etc.) and some small molecules (K^+^, Mg^2+^, etc.) (Bai et al., 2015; Xu et al., 2016). In response to antimicrobial agents, the release of cellular components can be used to assess the integrity of the bacterial cell membrane (Yi et al., 2014). The results indicated a dose-dependent release of intracellular components in BBR-treated *C. acnes*. Nucleic acids and proteins are essential for bacterial proliferation, the leakage of which will lead to bacterial death. Electrolytes such as K^+^ and Mg^2+^, which are crucial for maintaining energy levels, can cause changes in the structure of cell membranes and negatively impact cellular metabolism (Cowan, 2002; Gu et al., 2023). The rapid increase of K^+^ and Mg^2+^ in extracellular cells treated by BBR indicated that the permeability of the cell membrane increased, leading to leakage of intracellular components.

The cell walls of Gram-positive bacteria are characterized by a peptidoglycan (PG) layer about 30 nm thick. The Mur ligases play an indispensable role in the intracellular production of bacterial PG, which affords alluring targets for the development of antimicrobial drugs (Sink et al., 2008). The reactions catalyzed by these enzymes have been extensively studied over the past two decades. Concretely, the PG biosynthesis of Gram-positive bacteria is roughly divided into three steps: the first step in the cytoplasm, the second step at the cytoplasmic membrane, and the third step outside the cytoplasmic membrane (Zhou et al., 2022). In the bacterial cytoplasm, Mur ligase (MurA-F) adds amino acids stepwise to UDP-N-acetylmuramic acid (UDP-MurNAc) to form UDP-N-acetylcarbamoyl pentapeptide. Subsequently, MraY and MurG catalyze the membrane step of PG synthesis and produce lipids I and II, which are subsequently translocated by flippase from the inner to the outer membrane (Wachi et al., 1999; Lovering et al., 2012; Liu and Breukink, 2016).

The formation of the peptide stem required for the phase I reaction of bacterial PG biosynthesis primarily involves the four cytoplasmic enzymes (MurC, D, E, and F) of the Mur ligases (Sangshetti et al., 2017). Out of these four Mur ligases, MurC catalyzes ATP-dependent ligation of L-alanine (Ala) and UDP-MurNAc to form UDP-N-acetylmuramyl-L-alanine (UDP-MurNAc-L-Ala) (Isa, 2020). MurD causes the ATP-dependent addition of d-glutamic acid to UDP-MurNAc-L-Ala (Zheng et al., 2021). MurC and MurD are implicated in the synthesis of pentapeptide precursors. Thus, the suppression of MurC and MurD would lead to trouble with PG biosynthesis. The present study showed that the expression of *murC* and *murD* was simultaneously reduced when *C. acnes* was treated with BBR. It was revealed that BBR could interfere with stage I of PG biosynthesis in the cell wall of *C. acnes* by suppressing the expression of the *murC* and *murD* genes.

The consequent steps of PG synthesis occur at the interior surface of the plasma membrane of the cell, involving two enzymes, MraY and MurG, which are bound to the membrane (Liu and Breukink, 2016). MraY catalyzes the formation of lipid-I from UDP-MurNAc-pentapeptide (Henrich et al., 2016). In the presence of MurG, one molecule of UDP-GlcNAc is diverted to the MurNAc of lipid I to form lipid II, which is then transported by the flippase across the cytoplasmic membrane to the external environment (Ruiz, 2008; Galinier et al., 2023). The disaccharide pentapeptide portion of lipid-II acts as a substrate for PG synthase on the outside of the membrane, polymerizing and cross-linking to the PG layer. The integrity of the cell wall is compromised when the production of the PG layer is hindered, which causes cell rupture and death. The results of the study indicated that BBR could effectively down-regulate the expression of the *mraY* and *murG* genes in *C. acnes*, affecting lipid synthesis and disrupting the integrity of the PG layer.

Berberine exhibited *in vitro* strong antibacterial activity against *C. acnes*. When it was treated by BBR, *C. acnes* exhibited irreversible damage. The antimicrobial mechanism of BBR was associated with the inhibition of the expression of genes related to cell wall synthesis (*murC*, *murD*, *mraY*, and *murG*). On the one hand, it disrupted the cell wall and cell membrane, increasing the permeability of the cell wall and membrane, which in turn induced the loss of important intracellular components and ultimately led to the death of *C. acnes*. On the other hand, it perforated the cell wall and cytoplasmic membrane as well or accessed the cell after the cell structure had been disrupted, thereby decreasing the normal synthesis of substances that were necessary for bacterial growth.

On the basis of *in vitro* experiments, we have developed an animal model of *C. acnes* treatment, which is an important method for evaluating the anti-*C. acnes* and anti-inflammatory activity of BBR *in vivo*. In the pathogenesis of acne inflammation, *C. acnes* plays an influential initiating role through the production of chemokines (Huang et al., 2014). Our results indicate that intradermal attack of the mice ear by *C. acnes* causes a large infiltration of inflammatory cells, leading to ear edema, an inflammatory response, and the secretion of inflammatory mediators. IL-6 is a powerful inflammatory molecule with an endocrine role in acute or chronic inflammation (Gunter et al., 2020). The elevated expression of IL-6 has been found in acne-affected skin (Alestas et al., 2006). IL-1β is a potent inducer of inflammatory cytokine production (Cai et al., 2019). It has recently been shown that the active form of IL-1β is prevalent in inflamed acne lesions (Hsu et al., 2012). TNF-α is a crucial pro-inflammatory cytokine involved in the regulation of inflammatory responses and innate immunity (Lee et al., 2014; Lim et al., 2021). These inflammatory mediators have been implicated in exacerbating the inflammatory state of acne. The results indicated that BBR not only suppressed ear edema in *C. acnes*-induced mice but also improved the manifestation of inflammatory pathology in the auricle. Notably, BBR simultaneously inhibited the colonization of *C. acnes in vivo*, and the number of bacteria in mouse auricular tissues decreased significantly after BBR treatment, which was consistent with the results of the *in vitro* antimicrobial experiments. It shows that BBR has a promising *in vivo* antimicrobial and anti-inflammatory effect, which could improve acne inflammation by reducing the proliferation of *C. acnes*.

It should be noted that four strains of *C. acnes* were used to evaluate the *in vitro* activity of BBR, and only one strain (ATCC 11827) was carried out both *in vitro* and *in vivo* in our experiments. It is well known that the disease-associated strains display different characteristics (O’Neill et al., 2023). As for the prevalence of clinical drug resistance, it is better to involve some clinical strains and strains with different antibiotic-susceptible phenotypes to investigate the effects of BBR in further studies, which can offer more definitive data on the antibacterial properties of BBR.

In conclusion, our study suggests that BBR may exert its anti-*C. acnes* activity by interfering with the construction of *C. acnes* cell walls and cell membranes. Further studies revealed that BBR could suppress acne inflammation in mice. Taken together, BBR could be a prospective therapeutic strategy for the treatment of acne induced by *C. acnes*. In order to clarify the exact antimicrobial mechanism, we also need to investigate the effects of BBR on biofilm, energy metabolism, and virulence factors. These will be the main areas of investigation in subsequent research.

## Data availability statement

The original contributions presented in this study are included in this article/supplementary material, further inquiries can be directed to the corresponding authors.

## Ethics statement

The animal study was approved by the Chengdu University of Traditional Chinese Medicine Committee. The study was conducted in accordance with the local legislation and institutional requirements.

## Author contributions

LS: Data curation, Writing—original draft, Writing—review and editing. QY: Data curation, Writing—original draft, Writing—review and editing. FP: Conceptualization, Writing—review and editing. CS: Writing—review and editing, Conceptualization. DW: Data curation, Writing—original draft. LP: Formal analysis, Writing—original draft. FX: Writing—review and editing, Methodology. YT: Project administration, Resources, Writing—review and editing. CP: Conceptualization, Supervision, Writing—review and editing. QZ: Conceptualization, Formal analysis, Supervision, Writing—review and editing.
